# Safety of Pandemic (H1N1) 2009 Monovalent Vaccines in Taiwan: A Self-Controlled Case Series Study

**DOI:** 10.1371/journal.pone.0058827

**Published:** 2013-03-11

**Authors:** Wan-Ting Huang, Hsu-Wen Yang, Tzu-Lin Liao, Wan-Jen Wu, Shu-Er Yang, Yi-Chien Chih, Jen-Hsiang Chuang

**Affiliations:** 1 Epidemic Intelligence Center, Taiwan Centers for Disease Control, Taipei, Taiwan; 2 Fourth Division, Taiwan Centers for Disease Control, Taipei, Taiwan; Melbourne School of Population Health, Australia

## Abstract

In Taiwan, new H1N1 monovalent vaccines without adjuvant and with MF59® adjuvant were used in the nationwide vaccination campaign beginning on November 1, 2009. From November 2009 through February 2010, the authors identified recipients of H1N1 vaccines who were diagnosed with adverse events of special interest (AESIs) in a large-linked safety database, and used the self-controlled case series (SCCS) method to examine the risk of each AESI in the 0–42 days after H1N1 vaccination. Of the 3.5 million doses of H1N1 vaccines administered and captured in the linked database, the SCCS analysis of Guillain-Barré syndrome (GBS) found an incidence rate ratio of 3.81 (95% confidence interval 0.43–33.85) within 0–42 days after nonadjuvanted H1N1 vaccination and no cases after MF59®-adjuvanted H1N1 vaccination. The risks of other AESIs were, in general, not increased in any of the predefined postvaccination risk periods and age groups. The databases and infrastructure created for H1N1 vaccine safety evaluation may serve as a model for safety, effectiveness and coverage studies of licensed vaccines in Taiwan.

## Introduction

In June 2009, the World Health Organization declared the new influenza of swine-origin (H1N1) pandemic [1]. In Taiwan, the Advisory Committee on Immunization Practices recommended use of H1N1 vaccines in August 2009. New monovalent vaccines procured by the government and distributed to the public were inactivated vaccine without adjuvant (Adimmune Corporation, Taichung, Taiwan) for persons aged ≥1 year and MF59®-adjuvanted vaccine (Novartis Vaccines and Diagnostics, Sovicille, Italy) for persons ≥6 months [2]. The nationwide vaccination campaign started on November 1, 2009 at the peak of pandemic.

The trivalent seasonal influenza vaccine has a well-established safety record [3]–[6]. Multiple clinical trials assessed the immunogenicity and safety of the two H1N1 monovalent vaccines used in Taiwan, and data demonstrated that their safety profiles resembled those of the already approved seasonal influenza products [7]–[9]. However, because of the sample sizes of these trials, rare adverse events following immunization may not be detected until widespread use of the two vaccines in the population occurs. The importance of postlicensure safety assessment has also been demonstrated by the historical event of an increased risk of Guillain-Barré syndrome (GBS) with the receipt of the swine-origin influenza vaccine in 1976 [10].

The Taiwan government has implemented a multifaceted postlicensure safety surveillance strategy integral to its pandemic (H1N1) vaccination program [2]. The authors report the results of a population-based controlled study examining the risk of adverse events of special interest (AESIs) after H1N1 vaccination over a period of four months. Because the vaccinated and unvaccinated individuals are likely to differ in ways that are difficult to measure and control for, the analyses compare the risk of adverse events that occur during an exposed (risk) interval with events that occur during an unexposed (control) interval of the same vaccinated person who experience the AESI. This self-controlled case series (SCCS) analytic approach eliminates between-person confounding for a different inherent risk for these events regardless of vaccine receipt [11], [12].

## Materials and Methods

### Ethics Statement

This study was based on a prospective active surveillance program established by the H1N1 Central Epidemic Command Center (CECC) in response to potential safety concerns to the H1N1 vaccination program [2]. According to Article 17 of the Communicable Disease Control Act (http://dohlaw.doh.gov.tw/Chi/EngDownLoad.asp?msgid=171&file=efile1) and Article 3 of the Enforcement Regulations Governing the Central Epidemic Command Center (http://dohlaw.doh.gov.tw/Chi/EngDownLoad.asp?msgid=129&file=efile1) in Taiwan, data collection for this study was conducted during the H1N1 pandemic as part of the public health response functions of the CECC for surveillance purposes and therefore, this study did not require informed consent and was not reviewed by an institutional review board. This active surveillance program collected information on personal identifiers to link the H1N1 vaccination and adverse event datasets. The linked datasets were then stripped of identifiers and analyzed anonymously. This study was approved for publication by Taiwan Centers for Disease Control.

### Study Design

This study was conducted with automated data from a nationwide large-linked database (LLDB) developed by Taiwan Centers for Disease Control to evaluate the safety of H1N1 vaccine [2]. The LLDB linked data on demographics, H1N1 vaccinations, and *International Classification of Diseases, Ninth Revision, Clinical Modification* (ICD-9-CM) diagnosis codes using a unique identifier assigned to each resident. Subjects eligible for this study were those ≥6 months of age who received at least one dose of H1N1 vaccine and had an ICD-9-CM coded AESI from November 1, 2009 through February 28, 2010.

### Selection of Adverse Events

Taiwan’s National Health Insurance (NHI) is a social health insurance program administered by the government [13]. More than 99% of the 23 million Taiwan residents were enrolled in the program and 92% of all healthcare facilities in the country were contracted by the NHI system. Beginning January 2004, every insured person was issued a health insurance IC card. With the IC card, records of every patient visit were uploaded to the NHI IC Card Data Center daily.

The AESIs evaluated included GBS [3], [6], [10], [14], [15], demyelinating disease of the central nervous system (CNS) [3], [6], [14], convulsion [4]–[6], [14], encephalitis/myelitis [3]–[6], [14], Bell’s palsy [3], [6], [14], [16], acute hemorrhagic or ischemic stroke (“acute stroke”) [17], and idiopathic thrombocytopenia [6], [14]. These events were selected because they are serious, biologically plausible, and have been observed in published studies or passive surveillance as a consequence of influenza vaccination. Adverse events that occurred from November 1, 2009 through February 28, 2010 were identified using one or more ICD-9-CM diagnosis codes from the NHI IC Card Data Center database (Table 1). The ICD-9-CM codes were selected from certain medical settings (outpatient, inpatient, or emergency department) in an attempt to improve specificity by limiting the definitions to the relatively severe episodes. To further separate incident events from follow-up visits for pre-existing conditions, only the first adverse event of its category to occur during the four-month study period for that individual was counted, irrespective of the timing of vaccine administration.

**Table 1:** Adverse Events for Evaluation of Pandemic (H1N1) 2009 Monovalent Vaccine Safety.

Adverse Event	ICD-9-CM Code(s)	Medical Setting
Guillain-Barré syndrome	357.0	IP
Demyelinating disease of the CNS	340*, 341.0, 341.8, 341.9, 357.81, 377.30, 377.31, 377.32, 377.34, 377.39	IP
Convulsion	345*, 780.3, 780.31, 780.39	IP, ED
Encephalitis/myelitis^a^	323.5*, 323.6*, 323.8*, 323.9, 341.2*	IP, ED
Bell’s palsy	351.0	IP, ED, OP
Acute hemorrhagic or ischemic stroke	430*, 431*, 432*, 433.01, 433.11, 433.21, 433.31, 433.81, 433.91, 434*	IP, ED
Idiopathic thrombocytopenia^b^	287.3, 287.31, 287.5	IP, ED, OP

Abbreviation: CNS, central nervous system; ICD-9-CM, *International Classification of Disease, Ninth Revision, Clinical Modification*; IP, inpatient; ED, emergency department; OP, outpatient.

a Exclude if associated with any of the following within 0–7 days after encephalitis/myelitis diagnoses: 047.0, 047.1, 048, 049.0–049.8, 053*–056*, 058*.

b Exclude if associated with any of the following on the same day of idiopathic thrombocytopenia diagnoses: 140*–208*, 228*, 279*, 283*, 284*, 286.6, 570*, 571*, 742.59.

### Identification of Exposures

Unique billing codes had been developed by NHI to reimburse and differentiate between the nonadjuvanted and MF59®-adjuvanted H1N1 vaccinations. These billing codes were used to collect dates and types of vaccination, along with the personal identifier, gender, and date of birth of persons who received the H1N1 vaccine from the NHI IC Card Data Center databases. For persons who received the vaccine at nontraditional settings such as schools, workplaces, and large-scale vaccination stations, the information were extracted by registrars from clinical records and manually computerized into the vaccination data [2]. As of February 28, 2010, 3.5 million (62%) of the H1N1 vaccine doses administered to the Taiwan population data had been recorded in the exposure database (Table 2).

**Table 2:** Proportion of Pandemic (H1N1) 2009 Monovalent Vaccine Doses Administered That Were Recorded in the Study Database, by Age Group, November 1, 2009–February 28, 2010.

Age Group	Number of Doses Administered^a^(*n* = 5,656,110)	Number of Doses Recorded(*n* = 3,510,461)	Percent Recorded (%)
6 months to 6 years	685,289	561,461	(82)
7 to 17 years	2,991,018	1,838,783	(61)
≥18 years	1,979,803	1,110,217	(56)

a Data from National Influenza Vaccine Information System.

The exposure of interest was any H1N1 vaccinations from November 1, 2009 through February 28, 2010. For each study subject, the observation period began at time of first H1N1 vaccination and ended at the earliest of February 28, 2010 or death. The exposed (risk) period comprised of a predefined postvaccination 43 person-days (days 0 to 42), which, at maximum, would be two exposed time periods. The unexposed (control) period was person-days of the observation period outside the exposed periods. The prevaccination person-days between November 1, 2009 and the day before the start of the observation period were not included as the control period for AESIs to avoid concerns about bias due to indication or contraindication.

### Statistical Analyses

The SCCS analyses included only individuals who were diagnosed with an AESI and had received at least one dose of H1N1 vaccine during the observation period. In this case-only method, each subject’s observation period was divided into exposed (risk) and unexposed (control) person time. Incidence rates in risk time periods were compared to those in control time periods outside of the risk periods from the same individual. This within-subject comparison was similar to a stratified analysis with each subject as a unique stratum; therefore, subjects functioned as their own controls with implicit adjustment for measured and unmeasured confounders that did not vary over time [11].

The data were manipulated and analyzed using SAS®, version 9.2 (SAS Institute Inc., Cary, NC). Conditional Poisson regression models were used to estimate the incidence rate ratios (IRRs) and 95% confidence intervals (CIs) for each adverse event during the 0 to 42 days after vaccination by type of vaccine (nonadjuvanted and MF59®-adjuvanted). As each individual was in observation for less than four months, age was assumed not a time-varying confounder because the risk of AESIs was unlikely to vary significantly in such a relatively short time period. The AESIs were, however, stratified by age group for convulsion (0–5 and ≥6 years), Bell’s palsy (0–24 and ≥25 years), acute stroke (0–49 and ≥50 years), and idiopathic thrombocytopenia (0–24 and ≥25 years), to evaluate possible effect modification. The authors further calculated IRRs by risk subcategory for convulsion (postvaccination days 0–7 and 8–42) and other AESIs (days 0–14 and 15–42) after H1N1 vaccination, if at least three exposed cases were available for that subcategory. Stratified analyses were not performed for GBS and demyelinating disease of the CNS that occurred after nonadjuvanted vaccine, or GBS, demyelinating disease of the CNS, encephalitis/myelitis, and idiopathic thrombocytopenia after MF59®-adjuvanted vaccine because of the relative sparseness of cases in the observation periods.

## Results

**Figure 1:**
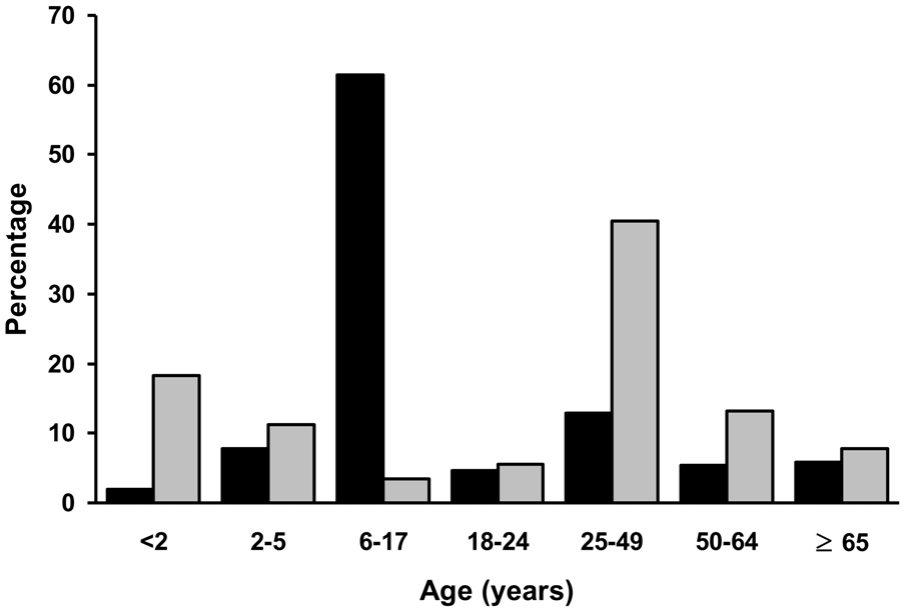
Proportion of pandemic (H1N1) 2009 monovalent vaccine doses administered, by age group and type of vaccine (without adjuvant, *black bars*; with MF59® adjuvant, *gray bars*), November 1, 2009–February 28, 2010.

A total of 3,510,461 doses of H1N1 vaccines were identified from the LLDB from November 1, 2009 through February 28, 2010, including 3,250,302 (93%) doses without adjuvant, 260,155 (7%) doses with MF59® adjuvant, and 4 (<1%) doses with unspecified vaccine type. The proportion of vaccine doses received by each age group varied (Figure 1). The majority (61%) of the nonadjuvanted monovalent vaccine were received by individuals 6–17 years of age (61%), whereas most (41%) of the MF59®-adjuvanted vaccine were received by individuals 25–49 years of age.

The recipients of H1N1 vaccine without adjuvant had 6 GBS, 12 demyelinating disease of the CNS, 1,103 convulsion, 15 encephalitis/myelitis, 414 Bell’s palsy, 849 acute stroke, and 59 idiopathic thrombocytopenia episodes identified during the study period of which 5 (83%) GBS, 5 (42%) demyelinating disease of the CNS, 497 (45%) convulsion, 10 (67%) encephalitis/myelitis, 223 (54%) Bell’s palsy, 421 (50%) acute stroke, and 32 (54%) idiopathic thrombocytopenia episodes occurred with 0–42 days after vaccination. The SCCS analyses found an increased but not statistically significant IRR of GBS (IRR 3.81, 95% CI 0.43–33.85) and encephalitis/myelitis (IRR 1.79, 95% CI 0.59–5.42) following receipt of the nonadjuvanted H1N1 vaccine. The risks of other AESIs after vaccination were generally not significantly increased in any of the predefined risk periods and age groups (Table 3). The IRR of convulsion for the 0–42 days after vaccination was significantly reduced; this effect was observed during the intervals 0–7 and 8–42 days and was independent of age (Table 3). For persons aged ≥50 years, there was a significant reduction in the risk of acute stroke at postvaccination 0–14 days (IRR 0.60, 95% CI 0.48–0.75).

**Table 3:** Association Between Adverse Events of Special Interest and Pandemic (H1N1) 2009 Monovalent Vaccine Without Adjuvant, by Age Group and Risk Interval, November 1, 2009–February 28, 2010.

Adverse Event	Age Group	Risk Period^a^	Number of Cases	Person Days	IRR	(95% CI)
Guillain-Barré syndrome	All	0–42	5	258	3.81	(0.43–33.85)
		Control	1	204	(Referent)	(Referent)
Demyelinating disease of the CNS	All	0–42	5	496	0.56	(0.16–1.93)
		Control	7	490	(Referent)	(Referent)
Convulsion	All	0–42	497	51,540	0.73	(0.64–0.82)
		Control	606	48,517	(Referent)	(Referent)
	0–5 years	0–7	34	3,752	0.67	(0.47–0.97)
		8–42	132	15,726	0.63	(0.50–0.78)
		Control	231	18,420	(Referent)	(Referent)
	≥6 years	0–7	58	6,088	0.72	(0.54–0.95)
		8–42	273	25,974	0.80	(0.68–0.94)
		Control	375	30,097	(Referent)	(Referent)
Encephalitis/myelitis	All	0–42	10	664	1.79	(0.59–5.42)
		Control	5	770	(Referent)	(Referent)
Bell’s palsy	All	0–42	223	18,198	1.03	(0.84–1.25)
		Control	191	16,666	(Referent)	(Referent)
	0–24 years	0–14	36	3,030	1.03	(0.70–1.52)
		15–42	56	5,539	0.88	(0.63–1.22)
		Control	96	8,417	(Referent)	(Referent)
	≥25 years	0–14	46	3,383	1.09	(0.76–1.56)
		15–42	85	6,246	1.13	(0.84–1.52)
		Control	95	8,249	(Referent)	(Referent)
Acute hemorrhagic or ischemic stroke	All	0–42	421	36,287	0.77	(0.67–0.89)
		Control	428	31,557	(Referent)	(Referent)
	0–49 years	0–14	16	1,965	0.58	(0.34–1.01)
		15–42	42	3,593	0.84	(0.56–1.24)
		Control	67	5,109	(Referent)	(Referent)
	≥50 years	0–14	104	10,836	0.60	(0.48–0.75)
		15–42	259	19,893	0.87	(0.74–1.03)
		Control	361	26,448	(Referent)	(Referent)
Idiopathic thrombocytopenia	All	0–42	32	2,705	1.09	(0.65–1.85)
		Control	27	2,566	(Referent)	(Referent)
	0–24 years	0–14	6	615	1.07	(0.41–2.85)
		15–42	16	1,101	1.59	(0.76–3.33)
		Control	14	1,560	(Referent)	(Referent)
	≥25 years	0–14	4	345	0.87	(0.28–2.67)
		15–42	6	644	0.70	(0.26–1.84)
		Control	13	1,006	(Referent)	(Referent)

Abbreviation: CNS, central nervous system; IRR, incidence rate ratio; CI, confidence interval.

a Risk (exposed) periods were days after each pandemic (H1N1) 2009 monovalent vaccination. Control (unexposed) periods were postvaccination time period outside the exposed periods.

No cases of GBS, demyelinating disease of the CNS, and encephalitis/myelitis were identified among recipients of MF59®-adjuvanted H1N1 vaccine. Table 4 described results from the various SCCS analyses for the exposed 0–42 days after vaccination with an MF59®-adjuvanted vaccine. The authors identified 162 convulsion, 34 Bell’s palsy, 94 acute stroke, and 9 idiopathic thrombocytopenia episodes among MF59®-adjuvanted vaccine recipients. Of these, 85 (52%) convulsion, 21 (62%) Bell’s palsy, 52 (55%) acute stroke, and 4 (44%) idiopathic thrombocytopenia episodes occurred with 0–42 days after vaccination. Immunization with the adjuvanted product was not significantly associated with increased risk of any AESI 0–42 days after vaccination. A significant reduction in the risk of convulsion for individuals aged 0–5 years in the 0–7 days (IRR 0.20, 95% CI 0.06–0.66) after vaccination was observed compared with the control period.

**Table 4:** Association Between Adverse Events of Special Interest^a^ and Pandemic (H1N1) 2009 Monovalent Vaccine Adjuvanted With MF59®, by Age Group and Risk Interval, November 1, 2009–February 28, 2010.

Adverse Event	Age Group	Risk Period^b^	Number of Cases	Person Days	IRR^c^	(95% CI)
Convulsion	All	0–42	85	7,803	0.89	(0.63–1.24)
		Control	77	7,246	(Referent)	(Referent)
	0–5 years	0–7	3	1,192	0.20	(0.06–0.66)
		8–42	54	4,894	0.90	(0.61–1.33)
		Control	63	5,634	(Referent)	(Referent)
	≥6 years	0–7	6	333	1.32	(0.45–3.86)
		8–42	22	1,384	1.46	(0.71–3.00)
		Control	14	1,612	(Referent)	(Referent)
Bell’s palsy	All	0–42	21	1,485	0.92	(0.43–2.00)
		Control	13	1,154	(Referent)	(Referent)
	≥25 years	0–14	8	435	1.24	(0.45–3.38)
		15–42	11	807	0.92	(0.35–2.35)
		Control	10	928	(Referent)	(Referent)
Acute hemorrhagic or ischemic stroke	All	0–42	52	3,974	1.02	(0.66–1.58)
		Control	42	4,029	(Referent)	(Referent)
	0–49 years	0–14	4	345	0.99	(0.31–3.22)
		15–42	7	636	0.96	(0.36–2.54)
		Control	12	1,227	(Referent)	(Referent)
	≥50 years	0–14	18	1,061	1.24	(0.66–2.31)
		15–42	23	1,932	0.93	(0.52–1.64)
		Control	30	2,802	(Referent)	(Referent)
Idiopathic thrombocytopenia	All	0–42	4	426	0.56	(0.13–2.36)
		Control	5	361	(Referent)	(Referent)

Abbreviation: CNS, central nervous system; IRR, incidence rate ratio; CI, confidence interval.

a No cases of Guillain-Barré syndrome, demyelinating disease of the CNS, and encephalitis/myelitis occurred among recipients of MF59®-adjuvanted vaccine.

b Risk (exposed) periods were days after each pandemic (H1N1) 2009 monovalent vaccination. Control (unexposed) periods were postvaccination time period outside the exposed periods.

c Analyses were performed if at least three exposed cases were available.

## Discussion

The authors evaluated AESIs in Taiwan during the time that H1N1 vaccine was used [2] and did not find statistically significant increased risk of any AESI in the six weeks after vaccination. This data on H1N1 vaccine safety were strengthened by the use of a nationwide healthcare database [13] to actively identify AESIs over a defined time period and by the large number of 3.5 million vaccine doses that was studied. The use of a vaccinated cases-only approach and a within-person SCCS comparison with postvaccination control period eliminates confounding factors that do not change during the study period, and reduces the bias due to incomplete doses capture and delayed or nonvaccination in cases with AESIs [11]. Also, the SCCS method is often more efficient in terms of power than other observation study designs and therefore, more precise estimates of effects can be made [18].

Although an elevated but nonsignificant risk of GBS after vaccination was observed, the small numbers of cases precluded any definitive conclusions on their association with H1N1 vaccines. The excess risks of approximately 1–2 cases per million doses administered in the U.S. and Canadian population-based surveillance for GBS after receipt of H1N1 vaccine [19]–[23] were similar to those observed after some previous seasonal influenza vaccines [15], but much lower than the 8.8 excess cases per million vaccinations observed with the use of 1976 swine influenza vaccine [10]. Antecedent respiratory infection or influenza-like illness is a recognized risk factor for GBS [24], [25] and an important confounder for the association between H1N1 vaccination and GBS, as confirmed in the European case-control study [26]. The majority of monovalent H1N1 vaccinations in Taiwan occurred during the period with circulation of pandemic (H1N1) 2009 viruses [2], [27]; however, the authors did not evaluate the effect of respiratory illness on the association between H1N1 vaccine and GBS in this SCCS study.

A signal of a potential association of H1N1 vaccines with Bell’s palsy during the 0–42 days after vaccination has been observed by a capture-recapture evaluation of passive safety surveillance data in Taiwan [28], in which the ratio of estimated compared with expected number of cases was 1.48 (95% CI 1.11–1.98). Results of this SCCS study, however, suggested that this signal may not represent a true increased risk of Bell’s palsy following immunization with either nonadjuvanted or MF59®-adjuvanted H1N1 vaccine. Bell’s palsy has been linked to seasonally varying infectious, immunologic, and vascular diseases [29]. Three published studies have assessed the association between H1N1 vaccine and Bell’s palsy [22], [30], [31]. In the Vaccine Safety Datalink study that found an excess risk of Bell’s palsy in adults aged ≥25 years receiving H1N1 vaccine in the self-controlled analysis, further assessment by using the case-centered approach to minimize confounding by seasonality observed no association [30]. In other studies, a small excess risk for Bell’s palsy was either observed only in comorbid persons who were targeted for H1N1 vaccination [31], or their evaluations did not control for seasonality of vaccination and disease [22], [31].

The findings that a reduction in the risk of AESI, particularly convulsion and acute stroke, was observed in some postvaccination time periods should be interpreted with caution. One possible explanation is that influenza infection is associated with major neurologic and vascular morbidities [17], [32], and vaccination averts H1N1 infection and associated morbidities. The authors, however, did not collect data on H1N1 infection and were unable to explore whether the beneficial effects of vaccination observed in this study were due to prevented influenza illness. An alternative explanation is confounding. Vaccination is often postponed for medical illness and individuals tend to receive a vaccine in a relatively healthy condition. The H1N1 vaccine may appear to be protective for any conditions that occur in the immediate time period after vaccination because fewer healthcare visits would be expected [33], [34]. For some AESIs evaluated in this study, the presence of this “healthy vaccinee effect” was suggested by a lower IRR for risk period that began at time of H1N1 vaccination compared with adjacent risk period that began at an interval of 8 or 15 days after vaccination. Also, a decrease in risk in this immediate postvaccination period was unlikely due to vaccination averting influenza disease and associated morbidity because recipients developed peak antibody protection against influenza infection two weeks after vaccination [7]–[9].

There were several additional limitations of the study. First, the adverse events were identified through ICD-9-CM codes and not validated against medical records. Use of ICD-9-CM codes would miss events that were not coded for, or the event status could be misclassified. The computerized data did not have information on the date of event onset but rather, the date of visit or admission was used. Insidious events could have variable lag time between symptom onset and medical attendance; their occurrences would be nondifferentially misclassified with respect to risk or control periods, which was likely to bias the IRR estimation toward unity. Second, because the capture of H1N1 vaccinations was only 62%, absence of H1N1 vaccination records in the LLDB, particularly for individuals ≥18 years of age and those received in nontraditional settings, did not necessarily indicate no vaccination [2]. The results from analyzing only cases with vaccination records in the LLDB might not be representative of H1N1 vaccine recipients. Furthermore, an individual’s postvaccination risk period could be misclassified as unexposed (control period) if a second dose of H1N1 vaccine was administered but not computerized into the exposure database. Third, the SCCS method controlled for confounders that did not vary over the observation period [11] but did not adjust for time-varying confounders. Other types of routine vaccines, for example, measles, mumps, and rubella vaccine and the 13-valent pneumococcal conjugate vaccine, were known to be associated with an increased risk of the prespecified adverse events (e.g., febrile convulsion or idiopathic thrombocytopenia) [35], [36], but their administrations were neither collected for evaluation as potential confounders in this study. Finally, even with a complete capture of 5.6 million H1N1 vaccine doses administered during the study period, there was limited statistical power to detect a very rare outcome such as GBS, if the excess risk associated with vaccine receipt is relatively small. The precise assessment of risk would require a larger population of subjects by pooling data through a regional or international collaboration.

In conclusion, the authors conducted safety evaluations of H1N1 monovalent vaccines in Taiwan and found no significantly increased risk of prespecified AESIs after receipt of either nonadjuvanted or MF59®-adjuvanted product. The possible association of GBS following nonadjuvanted H1N1 vaccine, although not statistically significant, was consistent with published studies in which a small excess risk was observed [19]–[21], [23]. As new vaccines are introduced and new recommendations are issued, it is desirable for countries to develop hypothesis-testing capacity to respond to potential safety concerns. The databases and infrastructures used in this H1N1 vaccine safety evaluation may serve as a model for safety, effectiveness and coverage studies of other licensed vaccines in Taiwan.
